# Detection of *Toxoplasma gondii* in Cord Blood Samples from Neonates in Tehran, Iran

**Published:** 2019-05

**Authors:** Moloud NAHVI, Saeedeh SHOJAEE, Hossein KESHAVARZ, Mahboobeh SALIMI, Mehdi MOHEBALI

**Affiliations:** Department of Medical Parasitology and Mycology, School of Public Health, Tehran University of Medical Sciences, Tehran, Iran

**Keywords:** *Toxoplasma gondii*, ELISA, Cord blood, IgG antibody, IgM antibody

## Abstract

**Background:**

Toxoplasmosis is an infectious disease caused by the coccidian protozoan parasite *Toxoplasma gondii*. The infection is life-threatening in congenital form because of transmission of the parasite from mother to fetus. In order to investigate the prevalence of congenital toxoplasmosis, the present study was performed for detection of IgG and IgM antibodies in cord blood samples of newborns by ELISA method in Tehran, Iran.

**Methods:**

This cross-sectional survey was carried out on 1000 cord blood samples collected from Shahid Mostafa Khomeini Hospital in Tehran, Iran in 2015. Sera were separated and evaluated for the presence of IgG and IgM antibodies against *T. gondii* by ELISA method. At the same time, whole cord blood samples were stored at −20 °C for complementary PCR test.

**Results:**

From 1000 cord blood serum samples 198 cases (19.8%) were positive for anti *T. gondii* IgG antibody. IgG positive samples were examined for IgM antibody, among them 1 sample had borderline levels of IgM antibody. PCR was performed for this sample but no positive result was seen.

**Conclusion:**

Although congenital toxoplasmosis is of importance, no acute form of infection was seen in pregnant women in this study.

## Introduction

*Toxoplasma gondii*, the obligate intracellular protozoa, is one of the most prevalent parasites in man and livestock and widely distributed around the world (1, 2). *T. gondii* is transmitted to humans by ingestion of oocysts in food, water or soil contaminated with cat’s feces, or by eating raw or undercooked meat containing tissue cysts (3, 4). Although the infection is benign, often self-limiting, and usually asymptomatic in immunocompetent individuals, it is life-threatening in immunocompromised patients (5, 6). Tachyzoites, the rapidly dividing stage of parasite could be transmitted from pregnant women to the developing fetus. Acute infection in untreated pregnant women may cause fetal transmission and congenital toxoplasmosis with complication outcomes in fetus (7). Diagnosis is critical in pregnancy and it is based on serological tests with detection of specific IgG and IgM antibodies. The prevalence of *T. gondii* infection in human populations is different from 4% to 92% in Korea and Brazil, respectively (8, 9).

Some *Toxoplasma* epidemiological studies have been conducted in Iran, for example in a recent systematic review the prevalence ranges from 18% to 70% has been reported (10).

Despite the large number of studies for detection of *T. gondii* infection in sera from Iran, there is no comprehensive and documented survey on cord blood samples in this country. To estimate the rate of congenital toxoplasmosis, this study was performed for detection of anti-*T. gondii* IgG and IgM antibodies in cord blood serum samples and PCR for IgM positive blood samples in Tehran, Iran.

## Materials and Methods

### Sampling

This cross-sectional study was performed on 1000 cord blood samples collected during 2015 from Shahid Mostafa Khomeini Hospital in Tehran, Iran. Sera were collected and kept frozen at −20 °C until use. In order to perform complementary PCR test, 1000 whole cord blood samples were collected from these cases and kept frozen too. The age of mother and gestational age was recorded in relevant questionnaires.

This study was approved by Ethical Committee of Tehran University of Medical Sciences, Tehran, Iran.

### Antigen preparation

Antigen preparation was performed (11). Briefly, tachyzoites of *T. gondii*, RH strain collected from peritoneal exudates of mice infected four days before, washed with phosphate buffer saline (P.B.S, pH 7.2) and sonicated for 10 sec in 15 times. After centrifugation at 14000g for 1 h at 4 °C, the supernatant was collected as soluble antigen then protein content determined by Bradford method (12).

### IgG ELISA

The microtiter 96 well plates were coated with 100 μl of carbonate buffer containing 5 μg/ml of protein and stored at −20 °C until use (13). For IgG ELISA, plates were washed with PBST (phosphate buffer saline, 5% Tween), then blocked with blocking buffer (skimmed milk 2.5% in PBST). After incubation and washing, 100μl of diluted sera (1:200) in PBST were added to each well.

After incubation and washing, 100μl of anti-human IgG conjugated with HRP (horse radish peroxidase) (DAKO, Denmark) in dilution of 1:500 with PBST was added to each well and then performed incubation and washing. Afterward, chromogenic substrate orthophenylene-diamidine (OPD) (Merck, Germany) was added to each well. After 15 min the enzymatic reaction was obvious and stopped by adding 20% sulfuric acid. Optical density (OD) was recorded at 492 nm with an automated ELISA reader (BIOTEC, LX800, USA).

### IgM ELISA

IgG positive samples were examined for anti-*T. gondii* IgM antibody. Detection of IgM antibody was performed by IgM-ELISA method. Briefly, *T. gondii* antigen-coated microtiter plate was prepared as described. Serum samples were diluted 1:20 in PBST and added to the antigen-coated wells, after incubation and washing, HRP conjugated anti-human IgM antibody (Dako, Denmark) was added. Afterward, incubation and washing, the substrate OPD (Merk, Germany) was added, and the OD of each well was recorded by an ELISA-reader (BIOTEC, LX800, USA) at 492 nm.

The optimal amount of antigen and dilution of serum and conjugated anti - *T. gondii* IgG and IgM antibodies were obtained by checkerboard method for each IgG and IgM antibody tests.

In each procedure, 50 random cord blood sera were tested by ELISA method and the cutoff was determined as the mean plus two times of the standard deviation of the absorbance readings acquired for random samples (X±2SD). The optical densities more and less than cutoff were considered as positive and negative.

### PCR Method

PCR was performed for samples with positive result in IgM ELISA. According to the PCR was performed for one sample with borderline IgM antibody (14). According to manufacturer’s instructions, the DNA was extracted from serum samples by PCR kit (QIA Gene amp DNA mini kit, Germany). The amplification of B1 gene was carried out with two sets of primers:

B1ToxoF 5′GGAACTGCATCCGTTCATGAG3′ B1ToxoR 5′TCTTTAAAGCGTTCGTGGTC3′. For amplification, 25μl of master mix (Ampliqon, Denmark), 4 μl of extracted DNA, 2μl of primer F and R, and 17 μl of distilled water were mixed by shaker. Then were centrifuged at 1000 g for 20 seconds.

The reaction was carried out in a thermocycler (PeQlab, England). After an initial denaturation at 95 °C for 10 min, 40 cycles were run, including denaturation (92 °C for 30 sec), annealing (55 °C for 50 sec), and extension (72 °C for 30 sec) and final extension at 72 °C for 7 min. PCR products and DNA ladder (Solis-Biodyne, Estonia) were electrophoresed in 1.5% agarose gel (Merck, Germany) and stained with ethidium bromide. The amplicons of 200 bp were visualized under UV illumination. In each time positive and negative controls were tested too. Negative control serum was negative for *T. gondii* IgG and IgM antibodies and positive control was DNA of tachyzoites of *T. gondii*, RH strain.

## Results

From 1000 cord blood serum samples, 198 cases (19.8%) were positive for anti-*T. gondii* IgG antibody. Prevalence of IgG anti-*Toxoplasma* antibody was higher in age group of 28–33 yr old. IgG positive samples were examined for IgM antibody which among them 1 sample had borderline result. PCR was performed for this sample; no DNA of the parasite was detected. The results of anti-*T. gondii* IgG antibody in cord blood serum samples in different mother’s age groups are summarized in Table 1.

**Table 1:** Frequency of anti-*T. gondii* IgG antibody in cord blood serum samples according to mother’s age

***Age groups(yr)***	***Positive N(%)***	***Negative N(%)***	***Total N(%)***
13–18	5(45.45)	6(54.54)	11
19–23	10(21.73)	36(78.26)	46
24–28	49(18.91)	210(81.08)	259
29–33	77(24.75)	234(75.24)	311
34–38	36(13.13)	238(86.86)	274
39–53	15(17.24)	72(82.75)	87
Missed	6(50)	6(50)	12

In Table 2 the frequency of anti-*T. gondii* IgG antibody in relation to the gestational ages are presented. The highest prevalence of infection is seen in 38 wk of pregnancy.

**Table 2:** Frequency of anti-*T. gondii* IgG antibody in cord blood serum samples according to the gestational age

***Gestation Period (wk)***	***Positive N(%)***	***Negative N(%)***	***Total N(%)***
33	1 (25)	3 (75)	4
34	1 (20)	4 (80)	5
35	3 (27.27)	8 (72.72)	11
36	16 (69.56)	7 (30.43)	23
37	20 (24.09)	63 (75.90)	83
38	74 (16.48)	375 (83.51)	449
39	64 (18.87)	275 (81.12)	339
40	8 (13.79	50 (86.20 )	58
41	2 (20)	8 (80)	10
Missed	9 (50)	9 (50)	18

## Discussion

Toxoplasmosis is one of the most prevalent parasitic infections with worldwide distribution. The most important form of infection is congenital toxoplasmosis. This condition occurs when the mother suffers from the infection during the pregnancy; depending on the age of the fetus it creates different effects. If a mother’s infection occurs during the first trimester of pregnancy, the fetal contamination ranges will be 15% to 20%, but damages of the fetus will be too severe and causes central nervous system defects, miscarriage, and fetal death. The risk of fetus infection in the third quarter is 65%, but in 90% of cases, it is without clinical signs (15).

The most common methods for laboratory diagnosis of *T. gondii* infection are serologic methods with determination of IgG and IgM antibodies in sera. In a pregnant woman with positive IgG and negative IgM antibody, the infection is chronic with no risk for the fetus. Positive IgM and a rising titer of IgG antibody indicate acute infection and the parasite can cross the placenta and infect the fetus. If both IgG and IgM antibodies are negative, mother has had no contact with *T. gondii* and is at risk of infection (7).

The distribution of toxoplasmosis in world with the highest rate of infection was seen in warm, moist areas and in countries such as Guatemala, Haiti, and El-Salvador with 100% of reported infection rate in adults. In cold regions, the amount of infection is reduced so in Alaska the lowest infection rate can be seen (16).

In a study from women referred to marriage consulting center of Arak, Iran, it was found the prevalence of 24.3% for anti-*Toxoplasma* IgG antibody (17). In Illam city, west of Iran was reported seroprevalence rate of 44.8% for *T. gondii* infection in pregnant women (18). The prevalence of 39.8% of anti-*T. gondii* antibody was reported in pregnant women from Gorgan city, north of Iran (19). The presence of IgG antibody in cord blood is related to crossing of maternal antibody via placenta to the fetus. There is no report of studies for detection of *T. gondii* infection in cord blood samples from Iran, so for the first time, this study was performed with 1000 serum samples of umbilical cord blood. In Brazil, from 1250 umbilical cord samples 1 serum was positive for IgM antibody (20). In Iraq, study on 300 umbilical cord blood samples, 105 positive IgG and 1 positive IgM was detected (21). In Finland, 42 out of 16,733 sera from pregnant women were suggestive for primary infection. After 12 months follow up congenital toxoplasmosis appeared in 4 cases (22). The prevalence of IgG antibody of *T. gondii* was determined in 28,247 serum samples belonging to 19,432 subjects of the area of Parma, Italy between 1987 and 1991, in which 2 cases of congenital toxoplasmosis were originated (23). In Australia, from 18,908 umbilical cord samples, 3 positive IgM sera were reported (24). Presence of IgG antibody in cord blood serum samples is related to crossing of maternal antibody via placenta to fetus but detection of IgM antibody could be related to immune responses of fetus to the infection.

As it mentioned in these studies the incidence of congenital toxoplasmosis is low worldwide. In our study from 1000 cord blood samples, 198 cases were positive for *T. gondii* IgG antibody in which 1 serum had borderline levels of IgM antibody with no positive result in PCR method. No sign of toxoplasmosis was observed in neonate.

## Conclusion

Although congenital toxoplasmosis is of importance for the fetus, no cases of acute infection were observed in pregnant women in this study.

## Ethical considerations

Ethical issues (Including plagiarism, informed consent, misconduct, data fabrication and/or falsification, double publication and/or submission, redundancy, etc.) have been completely observed by the authors.
